# Low Birth Weight, Small for Gestational Age and Preterm Births before and after the Economic Collapse in Iceland: A Population Based Cohort Study

**DOI:** 10.1371/journal.pone.0080499

**Published:** 2013-12-04

**Authors:** Védís Helga Eiríksdóttir, Tinna Laufey Ásgeirsdóttir, Ragnheiður Ingibjörg Bjarnadóttir, Robert Kaestner, Sven Cnattingius, Unnur Anna Valdimarsdóttir

**Affiliations:** 1 Centre of Public Health Sciences, University of Iceland, Reykjavik, Iceland; 2 Department of Economics, University of Iceland, Reykjavik, Iceland; 3 Department of Obstetrics and Gynecology, Landspitali University Hospital, Reykjavik, Iceland; 4 Department of Economics and the Institute of Government and Public Affairs, University of Illinois, Chicago, Illinois, United States of America; 5 Unit of Clinical Epidemiology, Department of Medicine, Karolinska Institutet, Solna, Stockholm, Sweden; 6 Centre of Public Health Sciences, University of Iceland, Reykjavik, Iceland; 7 Department of Epidemiology, Harvard School of Public Health, Boston, Massachusetts, United States; University of Tennessee Health Science Center, United States of America

## Abstract

**Objective:**

Infants born small for gestational age (SGA) or preterm have increased rates of perinatal morbidity and mortality. Stressful events have been suggested as potential contributors to preterm birth (PB) and low birth weight (LBW). We studied the effect of the 2008 economic collapse in Iceland on the risks of adverse birth outcomes.

**Study design:**

The study population constituted all Icelandic women giving birth to live-born singletons from January 1^st^ 2006 to December 31^st^ 2009. LBW infants were defined as those weighing <2500 grams at birth, PB infants as those born before 37 weeks of gestation and SGA as those with a birth weight for gestational age more than 2 standard deviations (SD's) below the mean according to the Swedish fetal growth curve. We used logistic regression analysis to estimate odds ratios [OR] and corresponding 95 percent confidence intervals [95% CI] of adverse birth outcomes by exposure to calendar time of the economic collapse, i.e. after October 6^th^ 2008.

**Results:**

Compared to the preceding period, we observed an increased adjusted odds in LBW-deliveries following the collapse (aOR = 1.24, 95% CI [1.02, 1.52]), particularly among infants born to mothers younger than 25 years (aOR = 1.85, 95% CI [1.25, 2.72]) and not working mothers (aOR = 1.61, 95% CI [1.10, 2.35]). Similarly, we found a tendency towards higher incidence of SGA-births (aOR = 1.14, 95% CI [0.86, 1.51]) particularly among children born to mothers younger than 25 years (aOR = 1.87, 95% CI [1.09, 3.23]) and not working mothers (aOR = 1.86, 95% CI [1.09, 3.17]). No change in risk of PB was observed. The increase of LBW was most distinct 6–9 months after the collapse.

**Conclusion:**

The results suggest an increase in risk of LBW shortly after the collapse of the Icelandic national economy. The increase in LBW seems to be driven by reduced fetal growth rate rather than shorter gestation.

## Introduction

Infants born small for gestational age or preterm have increased risks of perinatal morbidity and mortality [1] and of somatic diseases that can last throughout childhood and into adulthood [2], [3].

It is widely believed that a woman's emotional and psychological environment during the prenatal period can affect fetal development. Numerous studies have examined this hypothesis by obtaining associations between emotional and stressful life events during the prenatal period and adverse birth outcomes. However, results of these studies are inconsistent, with some studies reporting that adverse life events increase risks of poor pregnancy outcomes [4]–[14] and others reporting no association [15], [16] or the opposite [17].

Whether economic conditions during the prenatal period have adverse effects on infant health has been less investigated. Deheeja and Llers-Muney reported a reduced incidence of adverse birth outcomes during periods of high unemployment [18]. Margerison-Zilko et al. recently reported that unexpected economic contraction (measured as unexpectedly high monthly unemployment rate) early in pregnancy was associated with a decrease in birth weight [19]. Other studies have found either null associations [20], [21] or higher risks of low birth weight and neonatal mortality following recessions or involuntary unemployment [22]–[24].

On October 6^th^ 2008 the Icelandic prime minister informed the nation of an unusually swift and severe economic collapse in a dramatic manner and the government took over its three largest banks. The largely unforeseen collapse of the Icelandic economy with its associated rapid rise in unemployment and increase in household debt represents a potentially powerful stressor that may have adversely affected birth outcomes. Using the nationwide Medical Birth Registry, we examined the effect of the 2008 economic collapse in Iceland on infant health, as measured by low birth weight, preterm birth and small-for-gestational age birth.

## Materials and Methods

### Population

All Icelandic women registered in the National Icelandic Birth Registry from January 1^st^ 2006 to December 31^st^ 2009 (N = 16,616) were considered. We excluded women with multiple pregnancies (n = 298) and stillbirths (n = 47), leaving a total of 16,271 eligible women in the study.

### Outcome assessment

Information on birth weight in grams and gestational length in days was obtained from the Birth Registry. Low birth weight (LBW) was defined as less than 2,500 grams at birth and preterm birth (PB) as a delivery before 37 completed gestational weeks (259 days of gestation). For 16,228 births (>99.9%), length of gestation was based on ultrasound measurement before the 21^st^ week of gestation. In 7 pregnancies, gestational age could be estimated on the basis of last menstrual period. It could not be determined for 8 cases. Small-for-gestational age (SGA), a proxy for intrauterine growth restriction, was defined as infants with birth weight more than 2 standard deviations (SD) below the mean for gestational age according to the sex-specific Swedish fetal growth curve [25], which has been shown to be applicable for Icelandic fetuses [26]. Fetal growth rate index (Z scores) was also assessed by using methods previously described [25].

### Explanatory variables

The study period was dichotomized with pre-crisis period (“unexposed”), spanning from January 1^st^ 2006 to October 5^th^ 2008, and post-crisis period (“exposed”), spanning from October 6^th^ 2008 to December 31^st^ 2009. The pre- and post-crisis groups will hereafter be referred to as the unexposed (reference group) and the exposed group, respectively.

### Potential covariates

Information on covariates was obtained from the National Medical Birth Registry. Maternal characteristics obtained from the registry were: place of delivery; maternal age at delivery; parity (nulli-, primi- and multiparous); relationship status (mother cohabitating with father or not); employment status (employed or not employed (student/housewife/unemployed/on disablement benefit)); residence (living in the capital area or not). Maternal and infants' diseases were classified according to the International Classification of Diseases, tenth revision (ICD-10). Pregnancy-related diseases known to influence fetal growth included essential hypertension (ICD-10 code O10-O11), gestational hypertension and preeclampsia (ICD-10 codes O12-O15) and pre-gestational and gestational diabetes mellitus (ICD-10 codes O24.0-O24.2 and O24.4). Obstetric information obtained was: mode of delivery (vaginal or cesarean delivery), infants' sex, Apgar score at 5 minutes, vaginal induction of delivery (ICD-10 code O83.8), congenital malformations and chromosomal abnormalities (ICD-codes Q00-99) and early neonatal death (defined as death of a live-born infant within 7 days from birth). In order to account for seasonal variation of birth weight, the years were divided into four seasons and births occurring in the same season were grouped together.

### Statistical analysis

We calculated descriptive statistics for all maternal and obstetric characteristics as well as for the outcome variables, contrasting frequencies before and after the economic collapse. Differences in characteristics by exposure groups were explored using the Chi-square test for categorical variables and independent sample t-test was used for maternal age. Linear regression analysis was used for the continuous outcome variables gestational length and birth weight, adjusting for maternal age, parity and seasonality. One-way ANOVA tests with post-hoc Tukey's test were conducted to assess the homogeneity of birth weight and gestational length between seasons.

Logistic regression analyses were used to calculate adjusted odds ratios (aOR) and their 95% confidence intervals [CI's] for LBW, PB and SGA in the exposed period. In model I, adjustments were made for variables assessed as possible confounders: maternal age, parity and seasonality. In models II and III, we explored whether possible increased risks of adverse birth outcomes were mediated by other maternal factors or diseases during pregnancy all which, in fact, may have been influenced by the crisis. In model II, we therefore also adjusted for: hypertension and diabetes, and in model III we added relationship status, residence, employment status into the model. Analysis involving LBW and PB were also adjusted for infant's sex in models II and III. We used linear regression models to estimate changes in the fetal growth rate index across exposure categories. In order to explore further the risk of adverse birth outcomes among certain subgroups, we performed logistic regression analyses where we stratified for each of the following maternal characteristics: age, parity, relationship status, place of residence and employment status. The model used in the analyses was also adjusted for age (continuous), parity and seasonal variations (model I).

To further explore whether associations between PB, SGA and LBW differed depending on, when in gestation the collapse hit, we divided the study period into intervals of three months and compared those giving birth in a particular intervals in 2008 and 2009 with those giving birth in the same time intervals in 2006 and 2007. Each time interval in 2008 and 2009 averaged 1,050 births. In the comparison groups, the corresponding time intervals in 2006 and 2007 averaged 1,974 births.

In order to refine our exploration of potential crisis effects, we also used date of conception (instead of date of birth) to sort pregnancies according to exposure to the economic crisis. For example, women who conceived in the time period January – March 2008 (exposed to the crisis in the 3rd trimester) were grouped together and compared to women who became pregnant January – March 2005, 2006 and 2007 (unexposed) etc.

Further, in an attempt to detect a possible time-trend in LBW, SGA and PB that might falsely lead to measured exposure effects, we used linear regression analysis to calculate the monthly trend of each birth outcome in the pre-crisis period. This model was adjusted for maternal age and parity.

Additional analysis was conducted to examine the effect of the shock on fetuses that were in utero on the day of the collapse. The exposed group consisted only of those women who were pregnant on October 6^th^ 2008. Women, pregnant on October 6^th^ 2006 and 2007, were considered unexposed. Similar analysis was carried out to examine the effect of the crisis on women who became pregnant during the post-crisis period and gave birth in the last 7 months of 2009. The reference group consisted of women who became pregnant after October 6^th^ 2006 and gave birth the following year.

### Ethical considerations

The study was approved by the Icelandic National Bioethics Committee (VSNb2010050014/03.7), the Data Protection Authority (2010050499LSL/–) and the Directorate of Health (2010050296/5.6.1/HBS/hbs).

## Results

Among all 16,271 infants; 11,111 (68%) were in the unexposed group and 5,160 (32%) were in the exposed group. Table 1 and 2 present the maternal and obstetric characteristics by exposure status. Following the economic collapse, we observed a statistically significant increase in maternal age as well as a tendency towards higher parity. Compared to the pre-crisis period, mothers giving birth following the economic collapse were more likely to be single, and to have pregnancy-induced hypertension and gestational diabetes (table 1).

**Table 1:** Maternal characteristics during the study period, before and after Oct 6^th^ 2008.

Maternal characteristics	Category of characteristics	Precrisis (N = 11,111)		Postcrisis (N = 5,160)		*p-*value*
Mean age (SD)		29.01 (5.55)		29.24 (5.54)		0.016**
		Births, *n*	Births, %	Births, *n*	Births, %	
Age (year)	<25	2,454	22.09	1,055	20.45	0.036**
	25–34	6,734	60.61	3,160	61.24	
	≥35	1,923	17.31	945	18.31	
Parity	Nulliparous	4,324	38.92	1,966	38.10	0.072
	Primiparous	3,929	35.36	1,779	34.48	
	Multiparous	2,858	25.72	1,415	27.41	
Relationship status£	Cohabitating with father	9,422	86.38	4,182	84.18	<0.001***
	Single	1,485	13.62	786	15.82	
Place of residence¥	Rural	3,799	34.53	1,715	33.27	0.119
	Urban	7,203	65.47	3,438	66.73	
Employment statusβ	Working	8,247	75.23	3,783	74.48	0.312
	Not working	2,716	24.77	1,296	25.52	
Diabetes	No	10,783	97.05	4,953	95.99	0.001***
	Pre-existing	47	0.43	21	0.42	
	Gestational diabetes	281	2.53	186	3.60	
Hypertension	No	10,290	92.61	4,721	91.49	0.045**
	Pre-existing	151	1.36	82	1.59	
	Pregnancy-induced-hypertension	670	6.03	357	6.92	

£ Missing values n = 396 were excluded from analysis.

¥ Missing values n = 116 were excluded from analysis.

β Missing values n = 229 were excluded from analysis.

* *p*-values are based on Chi-square test, except for maternal age where independent sample t-test was used.

** Difference is statistically significant within p = 0.05.

*** Difference is statistically significant equal to or within p = 0.001.

**Table 2:** Obstetric characteristics during the study period, before and after Oct 6^th^ 2008.

Obstetric characteristics	Category of characteristics	Precrisis (N = 11,111)		Postcrisis (N = 5,160)		*p-*value*
Mean birth weight (g) (SD)		3,693.7 (569.38)		3,665.7 (570.31)		0.003
Mean gestational lengthΦ (days) (SD)		279.54 (12.10)		279.02 (11.91)		0.023
		Births, *n*	Births, %	Births, *n*	Births, %	
Mode of delivery	Vaginal	9,279	83.51	4,344	84.18	0.278
	Caecerian section	1,832	16.49	816	15.82	
Infant's gender€	Male	5,763	51.88	2,630	50.97	0.281
	Female	5,346	48.12	2,530	49.03	
Apgar 5min	7–10	10,868	97.82	5,050	97.87	0.850
	<7	242	2.18	110	2.13	
Congenital malformation	No	10,716	96.44	4,964	96.22	0.440
	Yes	395	3.56	196	3.78	
Early neonatal death (<7 days)	No	11,102	99.92	5,157	99.94	0.617
	Yes	9	0.08	3	0.06	
Low birth weight (<2500 g)	No	10,873	97.53	5,005	97.0	0.046
	Yes	274	2.47	155	3.0	
Small-for-gestational age	No	10,950	98.64	5,083	98.51	0.505
	Yes	151	1.36	77	1.49	
Preterm birth (<37 weeks)	No	10,619	95.64	4,918	95.31	0.342
	Yes	484	4.36	242	4.69	

Φ Missing values n = 8.

€ Missing values n = 2.

* *p*-values are based on Chi-square test, except for birth weight and gestational length where linear regression analysis, adjusted for maternal age, parity and seasonality was used. Significance level is 0.05.

The infants born in the period of the economic crisis weighed, on average, 28 grams less than infants in the reference group (table 2). There was also a small but statistically significant difference in mean gestational length between births in the exposed and unexposed periods. No differences were observed with respect to maternal residence, mode of delivery, sex of infants, Apgar score at 5 minutes, congenital malformation or early neonatal death. Post-hoc Tukey's test showed a statistically significant seasonal variation of both birth weight and gestational length in the pre-crisis period but not in post-crisis period (p<0.05 and p>0.05, respectively).

The rates of infants born with low birth weight (<2,500 grams) before and after the collapse were 2.5% and 3.0%, respectively. Table 3 shows the results for multivariate logistic regression analysis. When adjusting for maternal age, parity and seasonality (model I) we observed a statistically significant increase in the odds of LBW during the post-crisis period (aOR = 1.25 95% CI [1.02, 1.53]). When we further adjusted for other, possibly mediating variables (models II and III), the difference loses significance but remains elevated (aOR 1.17 95% CI [0.95, 1.45). Rates of preterm born infants were 4.3% before and 4.6% after the economic collapse. This difference was not statistically significant (table 3). Rates of SGA before and after the crisis were 1.4% and 1.5%, respectively. When applying logistic regression analysis, we found no significant association between time of crisis and risk of SGA (table 3) and aOR indicated relatively small differences (e.g., aOR 1.10).

**Table 3:** The separate and combined effect of covariates on the odds ratio of low birth weight, small for gestational age and preterm birth during the two study periods, before and after October 6^th^ 2008.

Covariates	Low birth weight (<2500 g)	Small for gestational age (SGA)	Preterm birth (<37 weeks)
	OR (95% CI)	OR (95% CI)	OR (95% CI)
**Model I** *	1.25 (1.02–1.53)	1.14 (0.86–1.51)	1.08 (0.92–1.26)
**Model II** **	1.22 (0.99–1.50)	1.11 (0.83–1.47)	1.06 (0.90–1.24)
**Model III** ***	1.17 (0.95–1.45)	1.09 (0.82–1.46)	1.03 (0.87–1.22)
**Crude**	1.23 (1.00–1.50)	1.10 (0.83–1.45)	1.08 (0.92–1.26)
**Seasonal variation**	1.24 (1.01–1.52)	1.13 (0.86–1.50)	1.08 (0.92–1.26)
**Maternal age**	1.23 (1.01–1.50)	1.10 (0.83–1.45)	1.08 (0.92–1.27)
**Parity**	1.23 (1.01–1.50)	1.10 (0.84–1.46)	1.08 (0.92–1.27)
**Sex**	1.22 (1.00–1.50)	α	1.08 (0.93–1.27)
**Diabetes**	1.22 (1.00–1.49)	1.10 (0.83–1.45)	1.07 (0.92–1.26)
**Hypertension**	1.20 (0.98–1.46)	1.06 (0.81–1.40)	1.07 (0.91–1.25)
**Relationship status**	1.19 (0.97–1.46)	1.10 (0.83–1.45)	1.08 (0.92–1.26)
**Place of residency**	1.21 (0.99–1.48)	1.10 (0.84–1.46)	1.06 (0.91–1.25)
**Employment status**	1.22 (0.99–1.49)	1.09 (0.83–1.45)	1.07 (0.91–1.26)

α SGA is inherently adjusted for infant's sex.

* Odds ratio adjusted for seasonal variation, maternal age and parity.

** Odds ratio adjusted for seasonal variation, maternal age, parity, sex, diabetes and hypertension.

*** Odds ratio adjusted for seasonal variation, maternal age, parity, sex, diabetes, hypertension, relationship status, place of residency and employment status.

Additional analysis was conducted to estimate the change in fetal growth rate index between pre- and post-crisis groups. Compared to the reference group, infants born in time of crisis, had a decreased fetal growth rate (*β* =  −0.004; *p* = 0.032). This decrease was particularly distinct for women giving birth in the time period April – June 2009 (*β* =  −0.015; *p* = 0.001).

**Figure 1:**
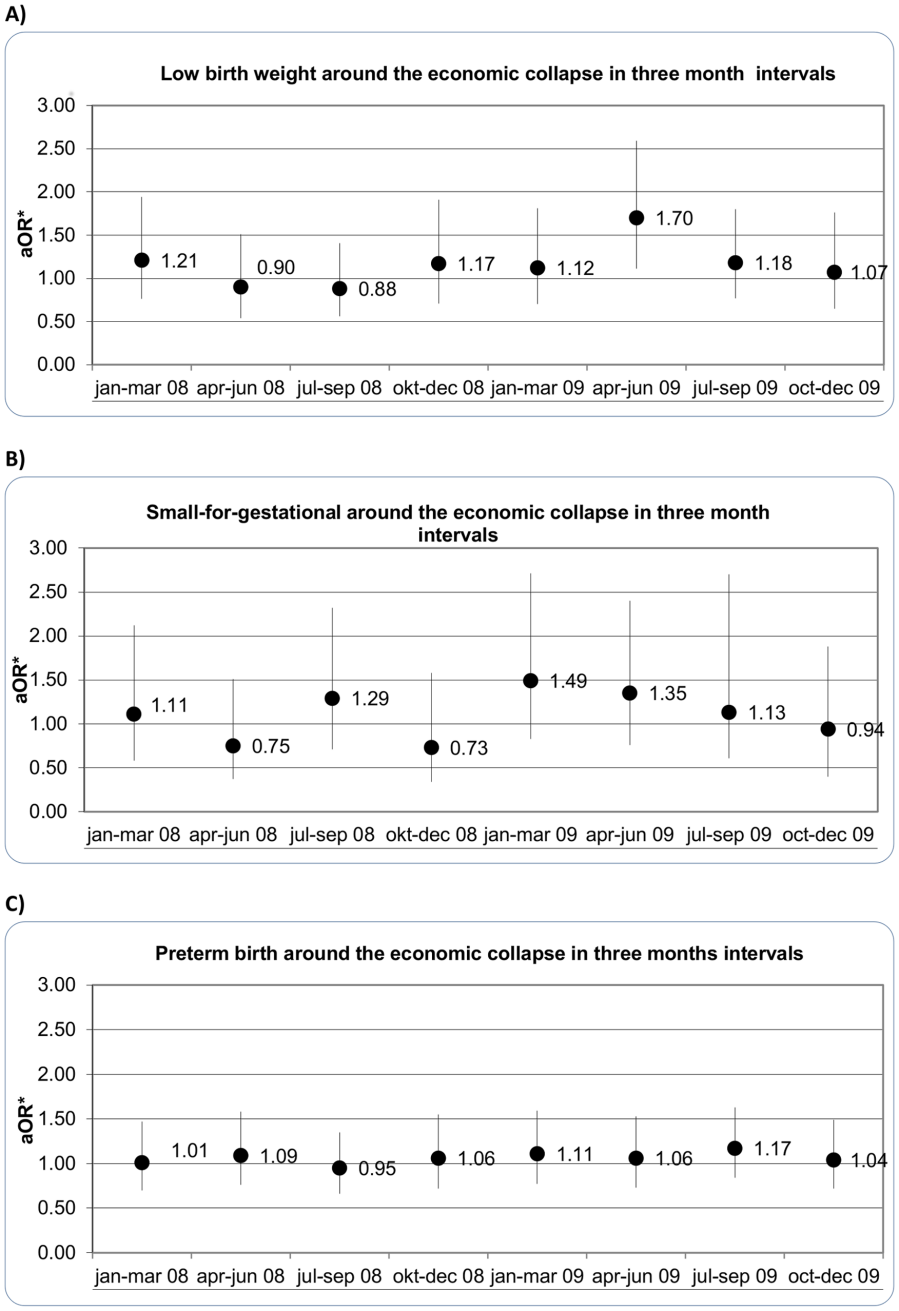
Odds ratio and 95% CI for (a) low birth weight, (b) small-for-gestational age and (c) preterm birth infants in Iceland for 8 three months intervals, prior to and after the economic collapse compared with the same intervals from each of two years before.

Figure 1 presents results from logistic regressions of LBW, SGA and PB around the economic collapse in three-month intervals, 3 intervals before the economic collapse (January 1^st^– October 5^th^ 2008) and 5 intervals after the collapse (October 6^th^ 2008– December 31^st^ 2009), using identical calendar times from 2006 and 2007 (combined) as reference periods. The first three comparisons are thus between before collapse time periods and can be viewed partly as falsification tests, for which we do not expect to see statistically significant results. After the economic collapse, we observed a statistically significant increased odds of LBW (aOR = 1.70, 95% CI [1.11, 2.59]) among women who were in their 1^st^ trimester when the crisis began, giving birth in the time interval April – June 2009, which is 6–9 months after the beginning of the crisis (figure 1a). A tendency towards increased odds of SGA was observed among women in their 2^nd^ and 1^st^ trimester during the onset of the crisis, giving birth in the time intervals January – March 2009 and April – June 2009, respectively (figure 1b). There were no associations observed between PB and stressors of the collapse in any of the three months intervals (figure 1c).

When classifying births according to date of conception rather than date of birth we obtained similar results. Women who conceived in the time period July – September 2008 and gave birth in April – June 2009 had increased risk of LBW and SGA but not of PB (appendix S1).

This pattern is also consistent with the results obtained from the analysis of fetuses on the day of the collapse, where a tendency towards increased risk of LBW and SGA deliveries was observed but not PB. Infants, conceived during the crisis, were not at increased risk of LBW, SGA or PB (appendix S2).

Table 4 presents multivariate adjusted odds ratios of LBW, SGA and PB during the crisis period stratified by age, parity, relationship status, place of residence and employment. Among mothers younger than 25 years, we observed a statistically significantly increased odds of giving birth to LBW and SGA infants during the crisis period as compared to before the crisis (aOR = 1.85, 95% CI [1.25, 2.72]; aOR = 1.87, 95% CI [1.09, 3.23], respectively). Similarly, if mothers were not working, corresponding post-collapse risks were increased (aOR = 1.61, 95% CI [1.10, 2.35]; aOR = 1.86, 95% CI [1.09, 3.17], respectively) compared to mothers not working prior to the collapse. Exposed mothers living outside the capital area also had increased odds of having a LBW compared to unexposed mothers (aOR = 1.53, 95% CI [1.07, 2.20]). There was no statistically significant difference in the odds of PB in any subgroups during vs. before the economic collapse and adjusted odds ratios were relatively small (close to 1).

**Table 4:** Adjusted odds ratio of low birth weight, small-for-gestational age and preterm birth during the study period, before and after Oct 6^th^ 2008, stratified by maternal characteristics.

Characteristics	Category of characteristics	aOR_LBW_ * (95% CI)	aOR_SGA_ * (95% CI)	aOR_PB_ * (95% CI)
**Age (year)**	<25	**1.85 (1.25–2.72)** **	**1.87 (1.09–3.23)** **	1.13 (0.81–1.58)
	25–34	1.05 (0.79–1.39)	0.81 (0.54–1.23)	1.06 (0.86–1.31)
	≥35	1.20 (0.78–1.87)	1.34 (0.76–2.37)	1.08 (0.75–1.55)
**Parity**	nulliparous	1.26 (0.95–1.68)	1.06 (0.73–1.55)	1.15 (0.91–1.45)
	primiparous	1.10 (0.74–1.64)	1.14 (0.63–2.06)	1.02 (0.74–1.39)
	multiparous	1.45 (0.95–2.23)	1.36 (0.74–2.50)	1.04 (0.75–1.43)
**Relationship status**	Cohabiting with father	1.15 (0.91–1.47)	1.18 (0.85–1.64)	1.02 (0.85–1.23)
	Single	1.36 (0.90–2.06)	1.01 (0.57–1.79)	1.20 (0.85–1.71)
**Place of resident**	rural	**1.53 (1.07–2.20)** **	1.33 (0.81–2.18)	0.94 (0.70–1.26)
	urban	1.11 (0.87–1.43)	1.06 (0.75–1.50)	1.12 (0.92–1.35)
**Employment status**	In work	1.13 (0.88–1.45)	0.94 (0.66–1.32)	1.06 (0.88–1.29)
	Not working	**1.61 (1.10–2.35)** **	**1.86 (1.09–3.17)** **	1.11 (0.81–1.51)

* OR adjusted for maternal age; parity and seasonal variation.

** Statistically significant difference between the time periods.

Finally, our linear regression analysis indicated no time-trends in LBW (F = 0.137; *p* = 0.714), SGA (F = 0.001; *p* = 0.972) and PB (F = 1.11; *p* = 0.301) in the pre-crisis period.

## Discussion

The results from this nationwide study indicate a decrease in mean birth weight as well as an increased rate of LBW deliveries in Iceland in the months following the economic collapse. This effect was mainly observed among relatively young mothers and mothers without a job. Women, in their 1^st^ trimester of pregnancy, at the time of the swift and dramatic collapse seemed most affected. This is in accordance with findings of Glynn et al., Lederman et al., Margerison-Zilko et al. and Mansour and Reed in their studies of major adverse life events effects on birth outcomes [6], [12], [19], [27]. Although, limited by small numbers, our findings suggest that the increase in LBW is driven by intrauterine growth restriction rather than shorter gestation. In an analysis of the unemployment crisis in Sweden in the 1990s, Bergmark and Palme identified subgroups that experienced greater welfare loss during the crisis. They found that young adults and single mothers (and immigrants), subgroups that already were socially and economically vulnerable, were particularly disadvantaged in terms of welfare resources [28]. These findings are in line with our findings which indicate that the increase in LBW and SGA births during the crisis period was considerable among young mothers (<25 years) and those without employment, compared with the same subgroups from before the collapse. Indeed, unemployment rates have been highest in this age group in Iceland during the crisis and rose up to 21% in the 2^nd^ quarter of 2009 and in 2010 [29]. Though, it should be acknowledged that due to the drastic rise in unemployment rates, the individuals constituting the group “not working” in the pre-crisis period might be different from those in the post-crisis period. Therefore, the positive association between the group not working and LBW should be interpreted with caution.

Dooley and Prause reported a decrease in birth weight of infants born to women who shifted from adequate employment to underemployment during pregnancy [24]. Furthermore, Catalano et al. found increased risk of very LBW infants among parents where the father was unemployed [30] and lastly, Jansen et al. found a decrease in mean birth weight among offspring of students and women receiving disability benefits [31].

It should be noted that the “not working” group in our study is heterogeneous, consisting of unemployed, disabled, housewives and students. This grouping may therefore not be comparable to other studies examining the effect of unemployment on birth outcomes. However, the largest group was students and it can be argued that being a student in Iceland at this time might have been a proxy for unemployment, as many of those who lost their jobs during the crisis subsequently went to school.

During the crisis, women living in rural areas were at higher risk of having a LBW than women living in urban areas. Since the impact of the crisis was in the beginning most severe for inhabitants living in the capital area and nearby areas, the opposite was expected. A possible explanation may be that the rural area category included a relatively densely populated area in the south-west part of Iceland, Suðurnes, which was hit especially hard by the economic crisis. Unemployment rate in Suðurnes was 13–14% in 2009, the highest in Iceland.

Hypertension has been identified as a risk factor for LBW, SGA and PB [32], [33]. The incidence of hypertension diagnosed during pregnancy did increase following the collapse but when hypertension was added to the models, the results did not indicate that the observed increases in LBW and SGA were altogether mediated via hypertension. Several other mechanisms may explain the observed association between the economic recession and increase in LBW/SGA. The economic collapse may have increased the stress levels among pregnant women causing direct physiological changes to the endocrine, immune and cardiovascular systems; changes that may affect the process of gestation to the worse [34], [35]. Furthermore, it is well recognized that stressful conditions, such as income shocks, may promote adverse health behaviors, e.g. smoking, drinking etc. [36], [37], thus acting as mediators between the stress caused by the economic collapse and the observed increase in LBW and SGA.

### Validity

This study leverages the National Medical Birth Registry to accomplish a population-based cohort study of all pregnant Icelandic women giving birth in Iceland in a four year time period. A multitude of information on the mother and child has been systematically collected to the registry since 1973, and this data collection is independent of exposure level, i.e. time of economic recession. Several measures were taken in order to further enhance the internal validity of this study. In order to make the cohort homogenous with regard to birth weight and length of gestation, we excluded all stillbirths and multiple gestations. Furthermore, our sample included only Icelandic women, as the literature indicates that risks of IUGR and PB may differ by ethnicity. Practically all pregnant women undertook ultrasound scanning around the 20^th^ week of pregnancy, and possible measurement errors of gestational length should be non-differential between the exposed and unexposed groups. Almost all (99%) births occur in hospitals or at local health clinics, resulting in accurate measurement of birth weight. The richness of information in the Medical Birth Registry allows us to control for most major confounding factors and our time trend analysis also indicates a somewhat stable rate of LBW before the economic collapse. Thus, we finally decided that changes occurring in most covariates (cohabitation, working–status, diabetes and hypertension) may actually be a consequence of the economic collapse and therefore in the causal chain between stress and LBW/SGA.

A limitation of this study is the lack of information on maternal smoking, alcohol, and nutritional habits during pregnancy. Smoking during pregnancy is causally associated with risk of LBW and IUGR [33], [38], [39]. Furthermore, some researchers have suggested that stressful circumstances are often alleviated by adverse health behavior, such as smoking [36], [37]. However, McClure et al. and Asgeirsdottir et al. report a significant reduction in the prevalence of smoking in Iceland between 2007 and 2009 among a representative cohort of 3755 Icelanders [40], [41]. Further, we did not have information on pre-pregnancy maternal weight. Low pre-pregnancy weight is associated with both SGA and LBW and high pre-pregnancy weight is associated with gestational diabetes and pregnancy-induced hypertensive diseases like preeclampsia, which often leads to SGA and PB [42]–[44]. Studies have indicated an increase in the prevalence of overweight and obesity among the Icelandic population over the last decades [45]. However, there are indications of a healthier lifestyle among Icelanders following the economic collapse in 2008, i.e. less consumption of fast food and sugar sweetened drinks [41]. Thus, it could be postulated that the short-term increase in LBW might be mediated by changes in body weight or smoking in the general population after the economic collapse. On the other hand, our previous study clearly indicates an increase in high stress levels among Icelandic women after the collapse [46] which strengthens our interpretation that the shock of the dramatic economic collapse may have contributed to the observed short-term increase in LBW. Nevertheless, further studies are needed to address if the effect of the economic crisis on LBW is mediated through altered behavior, exposure to heightened levels of stress hormones or both.

## Conclusion/Implication

The results of this study add important knowledge on how birth outcomes are affected when mothers are exposed to significant economic shocks such as the economic collapse that occurred practically overnight in Iceland. Our results suggest that the economic meltdown was an important stressor that increased the risk of LBW deliveries, especially for women in the 1^st^ trimester of pregnancy. The increase in LBW seemed to be driven by fetal growth restriction rather than by shortened gestation. The crisis appeared to have the largest effect on younger women (<25 years) and women who were not employed.

These findings suggest that the effect of the crisis on LBW was short lived, as women who were exposed during later periods of their pregnancy or who completed the pregnancy in the post-crisis period were relatively unaffected. However, further studies with longer follow-up are needed for definite conclusion, particularly to observe whether the effect for young and vulnerable women is persistent. The findings have implications for public health practice and clinical management of pregnant women, particularly young women and women in a vulnerable situation on the labor market.

## Supporting Information

Appendix S1 **Odds ratios and 95% CI for (a) low birth weight, (b) small-for-gestational age and (c) preterm birth infants, born to Icelandic women in time of economic collapse.** Women are grouped together by their date of conception into 5 three-months intervals; each period is contrasted with the combined time-intervals from the previous years.
(TIF)

Appendix S2 **a**) *Table 1*– The effect of covariates on the odds ratio of low birth weight, small for gestational age and preterm birth among women who were pregnant on October 6^th^ 2008 (n = 3130) compared with women who were pregnant on October 6^th^ in the two previous years (n = 6083). **b**) *Table 2*– The effect of covariates on the odds ratio of low birth weight, small for gestational age and preterm birth among women who became pregnant after October 6^th^ 2008 and gave birth in the last 6 months of 2009 (n = 2030) compared with women who became pregnant after October 6^th^ 2006and gave birth in the last 6 months of 2007 (n = 1898).
(DOCX)
