# Supplementary material for: Low Birth Weight, Small for Gestational Age and Preterm Births before and after the Economic Collapse in Iceland: A Population Based Cohort Study
Source: PLoS One. 2013 Dec 4;8(12):e80499. doi: 10.1371/journal.pone.0080499 (PMC3851132; doi:10.1371/journal.pone.0080499)
Supplement: Appendix S2 — a) Table 1– The effect of covariates on the odds ratio of low birth weight, small for gestational age and preterm birth among women who were pregnant on October 6th 2008 (n = 3130) compared with women who were pregnant on October 6th in the two previous years (n = 6083). b) Table 2– The effect of covariates on the odds ratio of low birth weight, small for gestational age and preterm birth among women who became pregnant after October 6th 2008 and gave birth in the last 6 months of 2009 (n = 2030) compared with women who became pregnant after October 6th 2006and gave birth in the last 6 months of 2007 (n = 1898). (DOCX) [file pone.0080499.s002.docx]

**Supplementary appendix 2**

**Table 1** - The effect of covariates on the odds ratio of low birth weight, small for gestational age and preterm birth among women who were

pregnant on October 6^th^ 2008 (n=3130) compared with women who were pregnant on October 6^th^ in the two previous years (n=6083).

| **Covariates** | **Low birth weight (<2500 g)** | **Small for gestational age (SGA)** | **Preterm birth (<37 weeks)** |
| --- | --- | --- | --- |
|  | **OR (95% CI)** | **OR (95% CI)** | **OR (95% CI)** |
| **Crude** | 1.25 (0.95 - 1.66) | 1.30 (0.90 - 1.88) | 1.05 (0.84 - 1.31) |
| **Model I*** | 1.25 (0.95 - 1.66) | 1.31 (0.91 - 1.90) | 1.05 (0.84 - 1.31) |
| **Model II**** | 1.23 (0.92 - 1.63) | 1.27 (0.88 - 1.84) | 1.03 (0.83 - 1.29) |
| **Model III***** | 1.18 (0.88 - 1.59) | 1.27 (0.87 - 1.85) | 1.01 (0.80 - 1.27) |

^α^ SGA is inherently adjusted for infant's sex

* Odds ratio adjusted for seasonal variation, maternal age and parity.

** Odds ratio adjusted for seasonal variation, maternal age, parity, sex, diabetes and hypertension.

*** Odds ratio adjusted for seasonal variation, maternal age, parity, sex, diabetes, hypertension, relationship status, place of residency and employment status.

**Table 2** - The effect of covariates on the odds ratio of low birth weight, small for gestational age and preterm birth among women who became pregnant after October 6^th^ 2008 and gave birth in the last 6 months of 2009 (n=2030) compared with women who became pregnant after October 6^th^ 2006and gave birth in the last 6 months of 2007 (n=1898).

| **Covariates** | **Low birth weight (<2500 g)** | **Small for gestational age (SGA)** | **Preterm birth (<37 weeks)** |
| --- | --- | --- | --- |
|  | **OR (95% CI)** | **OR (95% CI)** | **OR (95% CI)** |
| **Crude** | 1.05 (0.75 - 1.48) | 0.94 (0.56 - 1.57) | 1.06 (0.81 - 1.39) |
| **Model I*** | 1.06 (0.75 - 1.48) | 0.94 (0.56 - 1.58) | 1.07 (0.81 - 1.40) |
| **Model II**** | 1.05 (0.74 - 1.48) | 0.94 (0.54 - 1.59) | 1.06 (0.81 - 1.39) |
| **Model III***** | 1.08 (0.75 - 1.54) | 1.06 (0.60 - 1.86) | 1.10 (0.83 - 1.45) |

^α^ SGA is inherently adjusted for infant's sex

* Odds ratio adjusted for seasonal variation, maternal age and parity.

** Odds ratio adjusted for seasonal variation, maternal age, parity, sex, diabetes and hypertension.

*** Odds ratio adjusted for seasonal variation, maternal age, parity, sex, diabetes, hypertension, relationship status, place of residency and employment status
